# Multi-Angular Colorimetric Responses of Uni- and Omni-Directional Femtosecond Laser-Induced Periodic Surface Structures on Metals

**DOI:** 10.3390/nano11082010

**Published:** 2021-08-05

**Authors:** Taek-Yong Hwang, Yong-dae Kim, Jongweon Cho, Hai-Joong Lee, Hyo-Soo Lee, Byounghwak Lee

**Affiliations:** 1 Shape Manufacturing R&D Department, Korea Institute of Industrial Technology, Bucheon 14441, Korea; ydkim@kitech.re.kr; 2 Department of Physics, Myongji University, Yongin 17058, Korea; jwcho@mju.ac.kr; 3 Advanced Materials and Process R&D Department, Korea Institute of Industrial Technology, Incheon 21999, Korea; rookiehj@kitech.re.kr (H.-J.L.); todd3367@kitech.re.kr (H.-S.L.); 4 Department of Physics and Chemistry, Korea Military Academy, Seoul 01805, Korea

**Keywords:** laser-induced periodic surface structures (LIPSS), laser ablation, structural color

## Abstract

We investigated the colorimetric behaviors of metal surfaces with unidirectional low-spatial-frequency laser-induced periodic surface structures (UD-LSFLs) and omnidirectional LSFLs (OD-LSFLs) fabricated using femtosecond laser pulse irradiation. With the CIE standard illuminant D65, incident at −45°, we show that UD-LSFLs on metals transform polished metals to gonio-apparent materials with a unique behavior of colorimetric responses, depending on both the detection and rotation angles, whereas OD-LSFLs have nearly uniform gonio-apparent colors at each detection angle, regardless of their rotation. These colorimetric behaviors can be observed not only at the angles of diffraction but also near the angle of reflection, and we find that the power redistribution due to Rayleigh anomalies also plays an important role in the colorimetric responses of UD- and OD-LSFLs, in addition to diffraction.

## 1. Introduction

Nature creates a broad range of colors through periodic structures, pigments, and bioluminescence [1]. Periodic structures, when compared to the other two coloration mechanisms, present a unique capability to engineer colors through the modification of their periods and orientations as well as source and observer positions, since structural coloration by the periodic structures is rooted in diffraction [1,2].

In the past few decades, with femtosecond (fs) laser pulse irradiation, several types of quasi-periodic structures can be fabricated on metals [3,4,5,6,7,8,9]. Among these fs laser-induced periodic surface structures (LIPSSs), regarding structural coloration, low-spatial-frequency LIPSSs (LSFLs) have been popularly investigated because diffraction from LSFLs can be occurred in the entire range of visible wavelengths [4,5,9,10,11,12,13,14,15], and the viewing direction of the structural color can be adjusted easily by controlling the period and orientation of LSFLs with the incident angle and the polarization direction of fs laser pulses, respectively [7,8,13,15,16,17]. Moreover, LSFLs on metals with high hardness can be used to imprint themselves to the surface of soft metal such as Al [18], and this is promising for mass production. Accordingly, the structural coloration using LSFLs illustrates the broad applicability in industries.

Recently, we fabricated a new type of LSFL pattern, namely omnidirectional LSFLs (OD-LSFLs), expanding the viewing angle of structural colors by periodically ordering the orientation of LSFLs within the scanline [19]. Furthermore, compared to traditional LSFLs with a single orientation, unidirectional LSFLs (UD-LSFLs), OD-LSFLs uniformly distribute structural colors to all the azimuthal angles and show rotationally symmetric colorization under the normal illumination incidence [19].

The most notable applications for structural color materials are optical encryption and anti-counterfeiting due to their controllable optical properties [20]. As mentioned earlier, the structural colors from LSFLs can be easily altered by the polarization direction and wavelength of fs laser pulses. In particular, due to a quasi-periodic nature of LSFLs, their structural colors are unique in that it is exceedingly difficult to be duplicated through other classical means [4]. LSFLs are therefore uniquely suited for optical encryption and anti-counterfeiting [4,15]. To make use of UD- and OD-LSFLs for these applications, it is essential to understand how the colorimetric responses of UD- and OD-LSFLs change with their orientations and what mechanisms contribute to these colorimetric responses under specific positions of source and observer [4,15,20,21,22,23].

In this paper, we measure the modified colorimetric behavior of metal surfaces at multiple detection angles due to two types of LSFL patterns, UD-LSFLs and OD-LSFLs, produced by fs laser pulse irradiation with linear and periodically rotating polarizations. This work builds on our earlier study [19] and reports on angle-resolved distinctive characteristics of colors arising from quasi-periodic structural details at the nanometer scale. With the CIE standard illuminant D65, we show that both UD- and OD-LSFLs on metals can transform polished metals to gonio-apparent materials, and each has its unique behavior of colorimetric responses, depending on both the detection and rotation angles. In addition to diffraction, we find that the power redistribution due to Rayleigh anomalies also significantly affects the colorimetric responses of UD- and OD-LSFLs.

## 2. Materials and Methods

**Figure 1:**
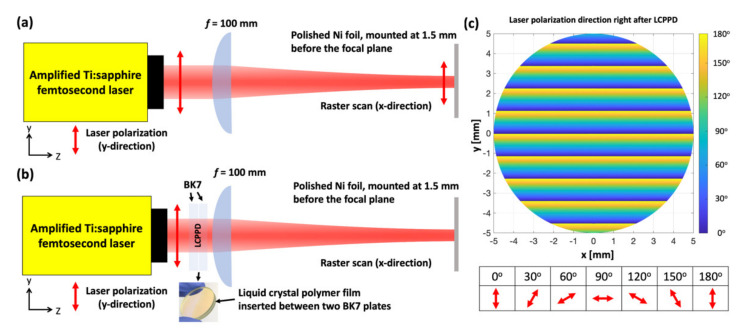
(**a**) Schematic of UD-LSFL fabrication in our experiments. The polarization direction of the fs laser pulses is in the *y*-direction. (**b**) Schematic of OD-LSFL fabrication in our experiments. The polarization direction of the fs laser pulses is manipulated by our liquid crystal polymer patterned depolarizer (LCPPD). (**c**) Calculated directions of polarization within the 1/e^2^ intensity radius of the fs laser pulses right after LCPPD. Angles denote the rotated angles of polarization direction due to LCPPD with respect to the *y*-direction.

**Figure 2:**
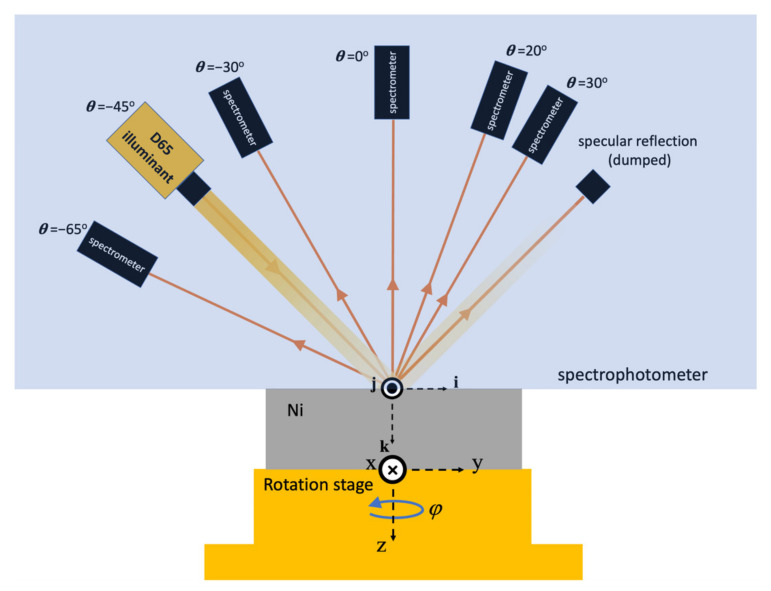
Configuration of the colorimetric measurements from the polished Ni, UD- and OD-LSFLs on Ni by using a spectrophotometer with detection angles of 30°, 20°, 0°, −30°, and −65°. The *ijk* axes are the space-fixed frame with their origin located in the center of the D65 illuminant spot at the sample surface. The *xyz* axes are the body-fixed frame, rotating with the Ni sample, and the sample rotation about the *z*-axis is indicated by *φ*, defined as 0° when the *x*-direction is antiparallel to the *j*-direction.

The laser employed in this experiment is an amplified Ti:sapphire fs laser system that generates 33.6-fs laser pulses with the maximum pulse energy of 1.2 mJ at a central wavelength and repetition rate of 800 nm and 5 kHz, respectively. At the fs laser output, the 1/e^2^ intensity radius is about 5 mm. We first prepare pure polycrystalline Ni (99.9%) with a thickness of 2 mm, and the surfaces are mechanically polished with 80-nm-grade colloidal silica. The average roughness of the polished Ni samples measured is 9.4 nm. To create UD-LSFLs on Ni, linearly polarized fs laser pulses are focused onto the samples with a 100 mm focal length plano-convex lens, as shown in Figure 1a. The surface of each sample is located at 1.5 mm before the focal plane. The 1/e^2^ intensity spot radius is about 80 μm at this defocused distance. To manipulate the polarization direction of fs laser pulses for the fabrication of OD-LSFLs, we insert a liquid crystal polymer patterned depolarizer (LCPPD) right before the focusing lens. A birefringent liquid crystal polymer film protected by two BK7 plates is used in the LCPPD, as shown in Figure 1b, and introduces an optical path difference of 380–430 nm between its fast and slow axes at our laser wavelength of 800 nm so that it nearly acts as a half-wave plate. Its fast axis also rotates 2° about the optical axis every 25 μm across the laser spot in the *y*-direction. Therefore, the polarization direction of fs laser pulses rotates periodically along the *y*-direction after LCPPD, and the period of the rotation by 180° is 1.125 mm, as visualized in Figure 1c. The 1/e^2^ intensity spot radius slightly increases to about 90 μm only in the *y*-direction at a defocused distance of 1.5 mm due to our LCPPD. Accordingly, the geometrical average of the spot radius, 85 μm, is used to calculate the laser fluence with our LCPPD. For colorimetric measurements with our spectrophotometer MA94 (X-Rite, Grand Rapids, MI, USA), UD- and OD-LSFLs are fabricated by raster scanning fs laser pulses with an area of 625 mm^2^ (25 mm × 25 mm), sufficiently covering the circular area required for measurements. The light source for illumination in our spectrophotometer is a gas-filled tungsten lamp, and the colorimetric response due to the light source is calibrated so that the D65 colorimetric illuminant with the CIE 10° 1964 standard observer is used in our experiments. The colorimetric responses of the samples are measured at five different angles of detection, −65°, −30°, 0°, 20°, and 30°, with our spectrophotometer at an illumination incident angle of −45°, as shown in Figure 2. Each sample is mounted on the rotation stage and rotated about the *z*-axis, while the spectrophotometer itself stands still. The rotation angle of the sample about the *z*-axis is indicated by *φ*, defined as 0° when the *x*-axis is antiparallel to the *j*-axis, depicted in Figure 2.

## 3. Results

**Figure 3:**
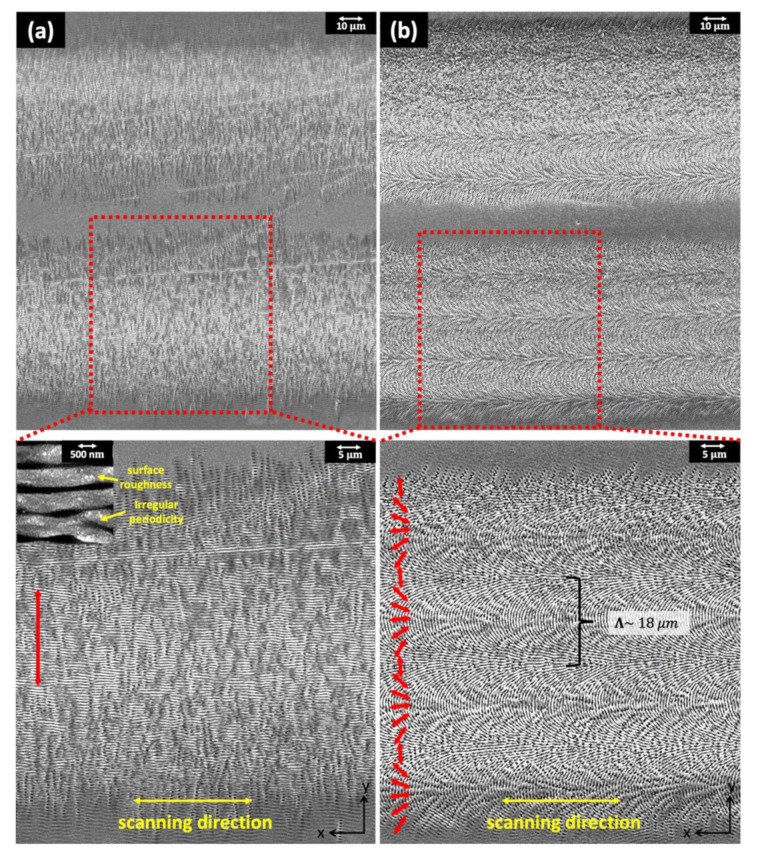
SEM images of (**a**) UD-LSFLs and (**b**) OD-LSFLs fabricated at a defocused distance of 1.5 mm. Double-headed red arrows indicate the grating vector (orientation) of UD- and OD-LSFLs and the polarization direction of fs laser pulses. The inset in (**a**) shows the surface roughness and irregular periodicity of UD-LSFLs on Ni.

Figure 3 shows the scanning electron microscope (SEM) images of UD- and OD-LSFLs, fabricated at normal incidence with laser fluences of 0.16 J/cm^2^ and 0.14 J/cm^2^ by raster scanning fs laser pulses with scanning speeds of 8 mm/s and 4 mm/s, respectively. Under these experimental conditions, the period of UD- and OD-LSFLs groove estimated by using the Fourier transform of the SEM images is about 0.64 ± 0.03 μm, and the modulation depth of their grooves is about 0.42 ± 0.15 μm, measured by a confocal laser scanning microscope. As described by the double-headed red arrows in Figure 3, the grating vectors of UD- and OD-LSFLs are determined by the polarization direction of the incident fs pulses, since the formation of LSFLs is mainly attributed to the inhomogeneous energy deposition due to the interference of the incident fs pulse with surface plasmon polaritons, excited by the incident pulse [2]. The thickness of the scanlines for both UD- and OD-LSFLs is in the range of 68–74 μm, while the distance between the scanlines is 90 μm. Accordingly, there exists a gap of 16–22 μm between the scanlines. This gap is deliberately inserted to eliminate any unwanted effects on structural coloration, resulting from the decreases in the reflectance [2] and period [24] of LSFLs by means of extra pulses of irradiation due to the overlapping of scanlines. Within a single scanline of OD-LSFLs, the number and period of LSFL orientation rotations by 180° are about 4 and 18 μm, respectively, and this is consistent with our previous work where the same defocused distance was used [19].

**Figure 4:**
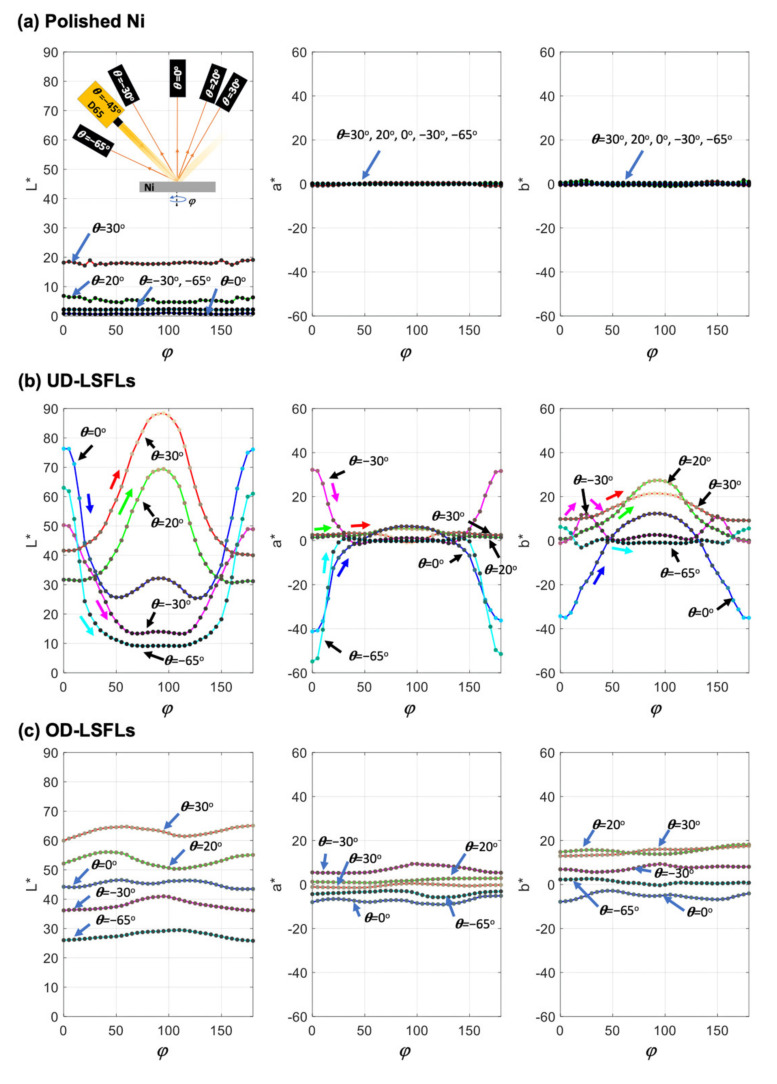
L*, a*, and b* in the CIELAB color space are measured in a rotation angle (*φ*) range of 0° to 180° on (**a**) the polished Ni surface, (**b**) UD-LSFLs on Ni, and (**c**) OD-LSFLs on Ni. Red, green, blue, magenta, and cyan solid lines are used to represent our colorimetric measurements at detection angles (*θ*) of 30°, 20°, 0°, −30°, and −65°, respectively. Each data point is represented by a small dot color-coded with actual angle-resolved true color. In (**b**), changing directions of L*, a*, and b* during an increase in *φ* from 0° to 90° are denoted by single-headed red, green, blue, magenta, and cyan arrows for detection angles of 30°, 20°, 0°, −30°, and −65°, respectively.

Figure 4 shows the dependence of structural colors from the three samples on the rotation and detection angles. The CIELAB color space is employed because it is device-independent [25] and the color and its difference can be easily quantified by three coordinates, L*, a*, and b* [26,27]. L* stands for the lightness value, brightness in the color space, and a* and b* represent colors changing from green to red and blue to yellow, respectively, as their values increase from negative to positive. The neutral gray color appears when a* and b* equal zero. The difference in color between two points, $\left(L_{1}^{*},\;a_{1}^{*},\;b_{1}^{*}\right)$ and $\left(L_{2}^{*},\;a_{2}^{*},\;b_{2}^{*}\right)$ in the CIELAB color space, ΔE, is defined as the Euclidian distance between these points, $\Delta E=\sqrt{{\left(L_{1}^{*}-L_{2}^{*}\right)}^{2}+{\left(a_{1}^{*}-a_{2}^{*}\right)}^{2}+{\left(b_{1}^{*}-b_{2}^{*}\right)}^{2}}$ [26,27], and is perceptible when ΔE>3.0 [25]. By considering the structural symmetry of UD- and OD-LSFLs in terms of their orientations, L*, a*, and b* values are measured in the rotation angle (*φ*) range of 180° at all detection angles (*θ*), and each data point is described by a small dot with actual true color, as shown in Figure 4.

On the polished Ni surface without LSFLs, as shown in Figure 4a, a* and b* are almost independent of the detection (*θ*) and rotation angles (*φ*), and located near zero. This indicates that the color of the polished sample is very close to neutral gray in our experiments. During the rotation of the sample, the maximum difference in color, $\Delta E_{max}$, within each detection angle is less than 3.0.

Figure 4b shows L*, a*, and b* for UD-LSFLs on Ni, and the changing directions of L*, a*, and b* during an increase in the rotation angle (*φ*) from 0° to 90° are represented by single-headed red, green, blue, magenta, and cyan arrows for detection angles of 30°, 20°, 0°, −30°, and −65°, respectively. Different from the polished Ni, a* and b* clearly change both with *θ* and *φ*, and the structural color can significantly deviate from neutral gray. For two backward detection angles of −30° and −65°, L*, |a*|, and |b*| tend to decrease as *φ* increases from 0° to 90°. Changes in b* with *φ* are a little bit complicated at *θ*= −30°, where b* reaches its maximum around *φ* ~ 25°. In the case of two forward detection angles of 20° and 30°, both L* and b* monotonically elevate as *φ* changes from 0° to 90°. Compared to L* and b*, the change in a* is relatively small at these forward detection angles. For *θ* = 0°, an increase in *φ* from 0° to 45° decreases L*, |a*| and |b*|, and the structural color from UD-LSFLs becomes a neutral gray color around *φ* = 45°. Then, L*, a*, and b* elevate all together when *φ* changes from 45° to 90°. During the rotation of the sample, the changes in color from UD-LSFLs are significantly large at all detection angles. The maximum values of measured color difference,$\Delta E_{max}$, are 49.8, 47.0, 80.1, 48.7, and 77.1 at *θ* = 30°, 20°, 0°, −30°, and −65°, respectively, during the change in *φ* from 0° to 90°. Due to the structural symmetry of UD-LSFLs and our color measurement configuration, the changes in the structural color of UD-LSFLs while *φ* increases from 0° to 90° are equivalent to the changes in *φ* from 180° to 90° at all detection angles, as shown in Figure 4b.

For OD-LSFLs, as shown in Figure 4c, the structural color changes mostly with the detection angle (*θ*), and appears to have a weak dependence on the rotation angle (*φ*) as compared to that for UD-LSFLs: during the rotation of OD-LSFLs, L*, a*, and b* are positioned within the variation range of those measured from UD-LSFLs within each detection angle. The maximum difference in color during the rotation of OD-LSFLs is less than 7.0 at each detection angle.

## 4. Discussion

As briefly mentioned earlier in Section 1, periodic structures can colorize material surfaces through diffraction. In fact, diffraction from periodic structures can be simply understood as the transfer of wavevector from the longitudinal direction to the transverse direction, where the amount of the transfer is the grating vector of periodic structures multiplied by integer numbers [28,29]. Assuming that our periodic structures are fabricated in the *ij* plane, as shown in Figure 2, the wavevector of diffracted light as functions of the wavelength of light (*λ*) and the rotation angle (*φ*) about the *z*-axis can be calculated by the following equation [29,30]:

(1) $$k_{\mathrm{diff}}\left(m\right)=\hat{i}\left(-k\sin\theta_{i}+m|K_{g}|\cos\varphi \right)+\hat{j}m|K_{g}|\sin\varphi \;-\hat{k}\sqrt{k^{2}-{\left(-k\sin\theta_{i}+m|K_{g}|\cos\varphi \right)}^{2}-{\left(m|K_{g}|\sin\varphi \right)}^{2}},$$

where $k_{\mathrm{diff}}$ and $K_{g}$ are the wavevectors of diffracted light and LSFLs, respectively, *k* is 2π/*λ*, $\left|K_{g}\right|$ is 2π/*d*, *d* is the period of one-dimensional periodic structures, and $\theta_{i}$ is the incident angle of illumination, −45° in our color measurement conditions. As described in Figure 2, $\hat{i}$, $\hat{j}$, and $\hat{k}$ are the unit vectors along the *ijk* axes of the space-fixed frame, and the origin of this system is located in the center of the D65 illuminant spot at the sample surface. In Equation (1), *m* represents the *m*th order of diffraction, available when the $\hat{k}$ component of $k_{\mathrm{diff}}$ is real.

In the case of the polished Ni sample, no deliberately fabricated periodic structures are present at its surface, and local roughness variations largely due to structural defects and polishing-induced surface scratches can effectively diffuse the reflected light. Because roughness-induced diffuse light plays a dominant role in its coloration, this scenario can account for our observation that L*, a*, and b* are nearly independent of *φ*, exhibiting neutral gray color at all detection angles, as shown in Figure 4a.

In the case of UD-LSFLs, however, diffraction comes into play on the colorimetric response of the surface due to a quasi-periodic nature of UD-LSFLs, and the surface roughness and quasi-periodicity of UD-LSFLs shown in the inset of Figure 3a diffuse the light near the diffraction and reflection angles.

**Figure 5:**
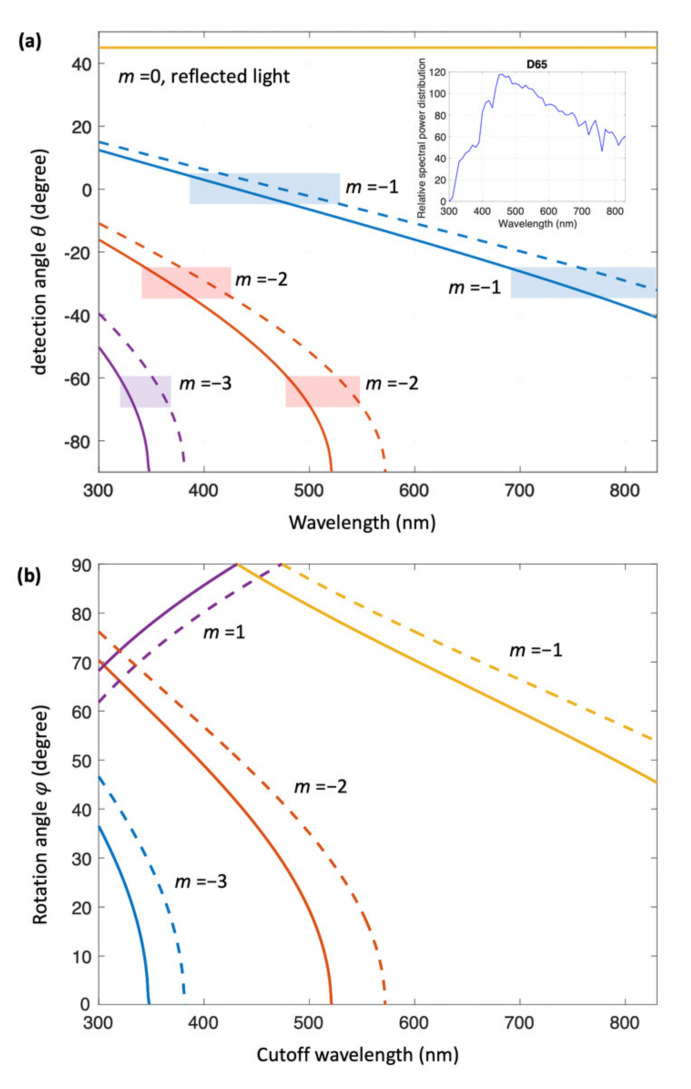
(**a**) Calculated *m*th order diffraction angles as a function of the wavelength of light. The ranges of the wavelength detected by three detection angles of 0°, −30°, and −65° are described by rectangular boxes filled with blue, red, and purple for *m* = −1, −2, and −3, respectively. For these boxed regions, the CIE 10° 1964 standard observer and a groove period range of 610–670 nm are considered. (**b**) Rotation angle *φ* versus the cutoff wavelengths for *m* = −3, −2, −1, and 1. Solid and dashed lines are used to describe the cases with groove periods of 610 and 670 nm, respectively.

With a UD-LSFL period range of 0.61 to 0.67 μm and the CIE 10° 1964 standard observer, the wavelength range of diffracted light can be obtained for *φ* = 0 at each detection angle with the help of Equation (1), as shown in Figure 5a. The −1st, −2nd, and −3rd orders of diffraction occur at our detection angles (*θ*) in the wavelength range of D65. However, any effects on color from the −3rd order of diffraction can be ignored, since the spectral luminous efficiency in photopic and scotopic vision is negligible at wavelengths below 380 nm [27].

Accordingly, for *θ* = −65°, the structural color of UD-LSFLs can be mainly attributed to the −2nd order of diffraction at *φ* = 0, and is close to bluish-green, the mixture of blue and green colors, where a dominant wavelength range of 480–552 nm is considered to primarily determine the perceived color. For detection angles of −30°, the −2nd and −1st orders of diffraction corresponding to wavelength ranges of 345–429 nm and 689–859 nm, both contribute to the color of UD-LSFLs respectively, leading the perceived color to be similar to purplish-pink. By increasing φ from 0° to 90°, the propagation direction of diffraction starts to have the *j* component, as described in Equation (1). Since our color measurements are restricted near the *ik* plane shown in Figure 2, the effect on L*, |a*|*,* and |b*| due to diffraction at our spectrometers should decrease while $\left|\sin\varphi \right|$ increases. As shown in Figure 4b, on UD-LSFLs for *θ* = −65° and −30°, the structural color of UD-LSFLs shifts toward neutral gray at φ = 90°, indicating that diffraction by UD-LSFLs mostly contributes to their color.

For forward detection angles of *θ* = 20° and 30°, the structural color of UD-LSFLs deviates from neutral gray at most of φ measured in our experiments, and the changes in L* and b* at these forward detection angles are quite different from those at the backward detection angles. According to Equation (1) and Figure 5, there is no diffracted light propagating toward these detection angles, and therefore it is expected that the diffused light due to surface roughness and the irregular periodicity of UD-LSFLs mainly affects the coloration of UD-LSFLs.

To better understand our observations, we consider Rayleigh or threshold anomalies [28,29]. Rayleigh anomalies explain the abrupt power changes to the propagating orders of diffracted lights and the reflected light at a cutoff wavelength (*λ*_c_) due to the power redistribution when a specific diffraction order disappears or appears with the changes in the wavelength and/or incident angle of light [28,29]. Considering no real part of the $\hat{k}$ component in $k_{\mathrm{diff}}$ in Equation (1), the cutoff wavelength (*λ*_c_) at all available diffraction orders (*m*) for the period range of UD-LSFLs, 0.61–0.67 μm, is calculated as a function of *φ* in Figure 5b. For *λ* > *λ*_c_, no diffraction occurs because the $\hat{k}$ component of $k_{\mathrm{diff}}$ is imaginary.

Based on our calculations, the cutoff wavelengths for *m* = −2 are in a wavelength range of 520–570 nm at φ = 0, and this range can be further broadened by the surface roughness and irregular periodicity of UD-LSFLs. Therefore, not all wavelength range of D65 illuminant in the visible spectral region is fully diffracted when *m* = −2, and some portion of power from the −2nd order of diffraction with the wavelengths longer than about 520 nm is redistributed into the diffused light, propagating toward detection angles (*θ*) of 20° and 30°. It follows that the structural color of UD-LSFLs becomes yellowish-pink, as a result of the contribution of red and some amount of yellow and green, whose corresponding wavelengths are mostly longer than about 550 nm in the visible spectral region, and the structural color deviates from the color of the polished sample, neutral gray. As *φ* increases until 45°, the cutoff wavelengths for *m* = −2 continuously decrease to about 416–458 nm shown in Figure 5b. More power from shorter wavelengths will be redistributed, and this leads to an increase in L*, as shown in Figure 4b. Additionally, the structural color of UD-LSFLs at these forward detection angles shifts toward orange-yellow, because the contribution of additional green and yellow with a small amount of blue to yellowish pink becomes larger with the decrease in the cutoff wavelengths for *m* = −2. This is equivalent to an increase in b^*^ since the contribution of more yellow than blue elevates b^*^ in the CIELAB color space. When we further increase *φ* to 90°, the cutoff wavelength for *m* = −2 can reach the wavelength below 400 nm, and the −2nd order of diffraction does not affect the structural color of UD-LSFLs anymore. Instead, the −1st order of diffraction comes into play by disappearing from red to blue. Consequently, the disappeared color from the −1st order of diffraction is continuously transferred toward our forward detection angles and keeps increasing L* and b^*^, the same reason for *φ* < 45°.

At *θ* = 0 °, the effects from both diffraction and Rayleigh anomalies are clearly observed on UD-LFSLs. When *φ* = 0°, the −1st order of diffraction with a wavelength range of 378–533 nm mostly determines the structural color of UD-LSFLs, greenish-blue. As *φ* increases, any effects due to the −1st order of diffraction reduce, due to our color measurement configuration limited near the plane of illumination incidence, and the structural color of UD-LSFLs shifts to neutral gray. However, once *φ* reaches 50° and further increases to 90°, Rayleigh anomalies clearly have an effect on the color of UD-LSFLs by disappearing the −1st order of diffraction as the cutoff wavelength decreases, and lead to the increases in L* and b^*^. More discussion about the angular-dependence of colorimetric responses shown in Figure 4b is available in Supplementary Materials.

In the case of OD-LSFLs, their structural color depends mainly on the detection angle, but does not change much on the rotation angle (*φ*). Moreover, it is not neutral gray, and varies within the colorimetric response variation of UD-LSFLs with *φ* at each detection angle, as shown in Figure 4c. This indicates that the structural color of OD-LSFLs is also subject to both diffraction and Rayleigh anomalies, and mostly is attributed to overall contribution from all the colorimetric responses of UD-LSFLs with the *φ* range of 0° to 180° since OD-LSFLs are UD-LSFLs with a periodic ordering of their orientations.

## 5. Conclusions

The colorimetric behaviors of the polished Ni, UD-LSFLs, and OD-LSFLs on Ni are investigated by using the CIE standard illuminant D65 at an illumination angle of −45°. We demonstrate that UD-LSFLs on metals can transform polished metals to gonio-apparent materials with their colorimetric responses that depend on the rotation angle of the sample, whereas the colorimetric response of OD-LSFLs changes only with the detection angle, and is nearly independent of the rotation angles. Furthermore, we find that these colorimetric responses of UD- and OD-LSFLs are attributed not only to diffraction but also to the wavelength-dependent power redistribution to the diffused light due to Rayleigh anomalies.

## Data Availability

The data presented in this study are available on request from the corresponding author.

## Supplementary Materials

The following are available online at https://www.mdpi.com/article/10.3390/nano11082010/s1, More discussion about the angular-dependence of colorimetric responses shown in Figure 4b is available.
