# Supplementary material for: Multi-Angular Colorimetric Responses of Uni- and Omni-Directional Femtosecond Laser-Induced Periodic Surface Structures on Metals
Source: Nanomaterials (Basel). 2021 Aug 5;11(8):2010. doi: 10.3390/nano11082010 (PMC8401486; doi:10.3390/nano11082010)
Supplement: Supplementary file 1 [file nanomaterials-11-02010-s001.zip › nanomaterials-1275251-supp.pdf]

Supplementary Materials

Multi-Angular Colorimetric Responses of Uni- and Omni-Directional Femtosecond Laser-Induced Periodic Surface Structures on Metals

Taek-Yong Hwang [1,*], Yong-dae Kim [1], Jongweon Cho [2], Hai-Joong Lee [3], Hyo-Soo Lee [3] and Byounghwak Lee [4,*]

1 Shape Manufacturing R&D Department, Korea Institute of Industrial Technology, Bucheon 14441, Korea; ydkim@kitech.re.kr
2 Department of Physics, Myongji University, Yongin 17058, Korea; jwcho@mju.ac.kr
3 Advanced Materials and Process R&D Department, Korea Institute of Industrial Technology, Incheon 21999, Korea; rookiehj@kitech.re.kr (H.-J.L.); todd3367@kitech.re.kr (H.-S.L.)
4 Department of Physics and Chemistry, Korea Military Academy, Seoul 01805, Korea
* Correspondence: taekyong@kitech.re.kr (T.-Y.H.); lebaiii@mnd.go.kr (B.L.)

It is worth mentioning that the distinct change in b^* with φ observed at $\theta = -30°$ in Figure 4b also relates both to diffraction and Rayleigh anomalies. In addition to the wavelength ranges of diffracted light for $\theta = -30°$ described in Figure 5a, the −1st order diffracted light with the wavelengths longer than the cutoff wavelength for $m = -2$ can be effectively diffused into our spectrometer due to the surface roughness and quasi-periodicity of UD-LSFLs, since the angle of –1st order diffraction at these wavelengths is the closest to our detection angle of -30° among those of all available diffraction orders. As discussed in the cases of $\theta = 20°$ and 30° on UD-LSFLs, the power redistribution due to Rayleigh anomalies consistently increases b^* with φ. Similarly, as φ changes from 0° to 25°, b^* continuously increases through more power redistribution to the diffused light. However, with a further increase in φ until 50°, the total amount of diffused light toward our detection angle shrinks much faster than the increasing redistributed power originating from Rayleigh anomalies due to our restricted color measurement in the *ik* plane, and b^* eventually reduces to near zero at this detection angle.
